# 

**Published:** 1826-02

**Authors:** 


### MONTHLY CATALOGUE OF MEDICAL BOOKS.

A Toxicological Chart; exhibiting at one view the Symptoms, Treatment, and Modes of Detecting the various Poisons, Mineral, Vegetable, and Animal: to which are added, Concise Directions for the Treatment of Suspended Animation. By WILLIAM STOWE, Member of the London College of Surgeons.—London.

Researches into the Nature and Treatment of Dropsy in the Brain, Chest, Abdomen, Ovarium, and Skin; in which a more correct and consistent Pathology of these Diseases is attempted to be Established, and a New and more Successful Method of Treating them, Recommended and Explained. By JOSEPH AYRE, M.D. Member of the College of Physicians, &c.—London, 1825.

The Anatomy of the Fœtal Brain; with a Comparative Exposition of its Structure in Animals. By FRÉDÉRIC TIEDEMANN, Professor in the University of Heidelberg, Member of the Academy of Sciences of Munich and Berlin, &c. &c. Translated from the French of A. J. L. JOURDAN, by WILLIAM BENNETT, M.D. To which are added, some late Observations on the Influence of the Sanguineous System over the Development of the Nervous System in general. Illustrated by 14 Engravings.—Edinburgh, 1826.

Further Observations on the Medicinal Leech; including a Reprint, from the Philosophical Transactions, of Two Memoirs, comprising Observations on the Hirudo Vulgaris, or Common Rivulet Leech; and on the H. Stagnalis and H. Complanata, now constituting the Genus Glossopora. With illustrative Engravings. By JAMES RAWLINS JOHNSON, M.D. F.R.S. F.L.S. Corresponding Member of the Society of the Faculty of Medicine, Paris; Honorary Member of the Physical and Natural History Society, Geneva; Extraordinary Member of the Royal Medical Society, Edinburgh; and late one of the Physicians to the Bristol Dispensary.—London, 1825.

An Enquiry into the Nature and Extent of the Rights, Privileges, and Powers, of the Faculty of Physicians and Surgeons of Glasgow; containing Commission from King James, Seal of Cause from the Magistrates of Glasgow, and Act of Parliament in favour of the Corporation of Chirurgeons and Barbers in Glasgow. By ALEXANDER ADAM, Surgeon; Member of the Faculty of Medicine, Glasgow; and of the Medical and Chirurgical Association, Greenock.—Glasgow, 1824.

Remarks on some parts of Mr. Calvert's Treatise on Diseases of the Rectum; namely, Hæmorrhoids, Simple Stricture, and Spasmodic Constriction of the Sphincter Ani. By W. WHITE, Member of the Royal College of Surgeons, London; Corresponding Member of the London Medical Society; and one of the Surgeons to the City Infirmary and Dispensary, Bath.—Bath, 1825.

Guilielmi Harveii Exercitationes de Motu Cordis et Sanguinis; quas Notis pauculis instruendas curavit THOMAS HINGSTON, M.D., Societatis Regiae Medicae Edinburgensis Socius, nunc ex Collegio Reginae Cantabrigiensi.—Edinburgi, 1824.

A Letter to Astley Cooper, Bart. F.R.S., Surgeon to the King, &c. &c., on certain Proceedings connected with the Establishment of an Anatomical and Surgical School, at Guy's Hospital. By J. H. GREEN, F.R.S., Professor of Anatomy to the Royal Academy; Professor of Anatomy and Surgery to the Royal College of Surgeons; Surgeon to St. Thomas's Hospital; and Lecturer on Anatomy and Surgery at that Hospital.—London, 1825.

A Treatise on the Physical and Medical Treatment of Children. By WILLIAM P. DEWEES, M.D. Member of the American Philosophical Society of Philadelphia, and of the Philadelphia Medical Society; Lecturer on Midwifery, &c. —Philadelphia, 1825.

Report of the Physician, laid before the General Court of the Hospital for Casual Small-Pox and Vaccination, at St. Pancras; held December 1, 1825.—London, 1826.

De Gastromalachia et Gastropathia Infantum. Auctore F. X. RAMISCH.—Prague, 1824.

Anatomie du Cerveau dans les quatre Classes d'Animaux Vertebrés, comparée especialement a celle du Cerveau de l'Homme. Par LAURENCET, de Lyon; avec Planches. Paris, 1825.

Manuel du Pharmacien; ou, Precis Elementaire de Pharmacie, par A. CHEVALLIER, &c. &c. et par P. SAT, de Lyon. Tom. I. et II.—Paris, 1825.

*(Others have been received, but are omitted from want of room.)*

### NOTICE TO CORRESPONDENTS.

*We beg to acknowledge the receipt of* Dr. Marshall Hall's *polite Letter. We shall be happy to hear from him again.*

*We thank* Dr. Dewees *for his Letter, and his Work on the* Diseases of Children. *We have analysed it in our present Number.*

*We regret very much that we cannot give insertion to the* Observations on Hemorrhage. *They are, in general, correct; but the Author will find his views anticipated in most elementary works upon the subject: they are true, but not new.*

*The Paper of* ’ΑΔΕΛΦΟΣ *is not fitted for this Journal.* We *cannot become the advocates of the Court of Examiners: the first page of the Wrapper contains an insuperable obstacle.*

*The Communication containing Remarks on* Dr. Davis's *Work on* Operative Midwifery *does not appear to us sufficiently practical or important for insertion.*

*The Case of* Intestinal Hemorrhage *is not fit for publication.*

*The same applies to the* Remarks on Probes and Sounds.

*The Communications of* Mr. Brown *and* Mr. Wade *have been received.*

